# MiR-124-3p/B4GALT1 axis plays an important role in SOCS3-regulated growth and chemo-sensitivity of CML

**DOI:** 10.1186/s13045-016-0300-3

**Published:** 2016-08-12

**Authors:** Yu-xiao Liu, Li Wang, Wen-jia Liu, Hai-tao Zhang, Jing-hui Xue, Zhi-wen Zhang, Chun-ji Gao

**Affiliations:** 1 Department of Neurosurgery, The First Affiliated Hospital of Chinese PLA General Hospital, 51 Fushi Road, Beijing, 100048 People’s Republic of China; 2 Department of Hematology, Chinese PLA General Hospital, Laoshan Branch, No. 401, Qingdao, 266100 People’s Republic of China; 3 Department of Hematology, Chinese PLA General Hospital, 28 Fuxing Road, Beijing, 100853 People’s Republic of China; 4 Genetic Laboratory of Development and Diseases, Beijing Institute of Biotechnology, Beijing, 100071 People’s Republic of China

**Keywords:** SOCS3, miR-124, B4GALT1, Leukemogenesis, Chemo-sensitivity

## Abstract

**Background:**

Abnormal expression of SOCS3 has been implicated in myeloproliferative neoplasms, but the role of SOCS3 in the pathogenesis of leukemia remains largely unknown. Here, we examined the function of SOCS3 in the growth and chemo-sensitivity of chronic myeloid leukemia (CML) and explored the involved mechanisms.

**Methods:**

Expression levels of SOCS3 in several leukemia cell lines and bone marrow mononuclear cells (BMNCs) from CML patients were determined using quantitative real-time PCR (qPCR) and Western blotting (WB). The roles of SOCS3 in the proliferation, apoptosis, and drug resistance of CML cells were examined by clonogenic progenitor cell assay, flow cytometry, and CCK-8 assay. A detailed analysis of the underlying mechanism of SOCS3 in K562 cells was performed using the Human HT-12 v4 Expression BeadChip, which has more than 48000 gene probes including 600 microRNAs (miRNA) probes. The correlation between the mRNA expression of SOCS3 and miR-124-3p in BMNCs from 30 CML patients was tested by qPCR and analyzed by Pearson correlation and linear regression analysis. The potential target of miR-124-3p in CML cells was explored using the luciferase reporter assay, qPCR, and WB. The effect of SOCS3 on the miR-124-3p/B4GALT1 axis was investigated by qPCR, WB, CCK-8 assay, and tumorigenicity assays in nude mice.

**Results:**

SOCS3 was down-regulated in CML cell lines and most of BMNCs from CML patients, and the expression level of SOCS3 was associated with the inhibition of cell proliferation and drug resistance of CML cells. Over-expression of SOCS3 in K562 cells inhibited the expression of leukemia-specific genes and promoted the expression of some miRNAs, among which miR-124-3p was the highest. SOCS3 over-expression enhanced the expression of miR-124-3p and vice versa. The mRNA expression of miR-124-3p and SOCS3 in BMNCs from 30 CML patients was positively correlated. Consistently, the tumor suppressing effects of SOCS3 were partially neutralized by the miR-124-3p inhibitor. B4GALT1 was downstream of miR-124-3p and regulated by SOCS3/miR-124-3p in vitro. Furthermore, SOCS3 over-expression could inhibit the growth and B4GALT expression of K562 cells in vivo.

**Conclusions:**

SOCS3/miR-124-3p/B4GALT1 axis plays an important role in the pathogenesis of CML.

**Electronic supplementary material:**

The online version of this article (doi:10.1186/s13045-016-0300-3) contains supplementary material, which is available to authorized users.

## Background

Suppressor of cytokine signaling (SOCS) is a protein family of eight members (SOCS1–7 and CIS) that form a classical negative feedback system to regulate cytokine signal transduction [1]. By regulating the cytokine-driven STAT activation pathway, SOCS3 likely has an important role in development, allergic responses, and tumorigenesis [2–4]. Methylation of the SOCS3 promoter and reduced gene expression of SOCS3 have been documented in numerous tumors such as breast cancer, lung cancer, and liver cancer [5–7]. SOCS3 is highly conserved among vertebrates and has been considered to be a transcriptional regulator in the hematopoietic system. SOCS3^−/−^ mice die in utero because of fetal liver erythrocytosis, and over-expression of SOCS3 blocks fetal liver erythrocytosis, implying that SOCS3 plays a critical role in the negative regulation of hematopoiesis [8, 9].

Evidence suggests a potential role of SOCS3 in the pathogenesis of leukemia. For example, Capello et al revealed that inactivation of SOCS3 was frequent in Ph-negative chronic myeloproliferative disorders (CMPD) [10]. Al-Jamal et al. reported that down-regulation of SOCS3 was involved in the resistance of CML cells to imatinib [11]. However, the exact function of SOCS3 in hematological malignancies remains unclear. A better understanding of the function and underlying molecular mechanisms of SOCS3 will contribute to the precision medicine in the field of CML.

In a previous study, we showed that SOCS3 was important for lineage commitment of hematopoietic stem cells to erythroid cells. SOCS3 knock-down increased the expression of multiple erythroid-specific genes and inhibited the expression of genes controlling lymphoid differentiation [12]. In this study, we wanted to investigate the contribution of SOCS3 in pathogenesis of CML and further understand the potential underlying mechanisms of SOCS3. Previous studies suggested dysregulation of microRNA (miRNA) networks had been implicated in hematological malignancies, for example miR-29a/29b dysregulation played an important role in myeloid leukemogenesis [13]. So, understanding of miRNA biology in carcinogenesis could possibly pave novel routes for anti-cancer therapy [14, 15]. Here, we found that over-expression of SOCS3 in CML cells induced a transcriptional program enriched for leukemia suppression factors, including some miRNAs. For example, miR-124-3p was obviously up-regulated by SOCS3 over-expression. In turn, alterations of miR-124-3p expression levels influenced the effect of SOCS3 on CML cells. Furthermore, we confirmed that B4GALT1, a multidrug resistance gene, was the target gene of the SOCS3/miR-124-3p axis. These findings suggested the presence of a dysregulated molecular network involving SOCS3, miR-124-3p, and B4GALT1, which may provide novel insights into tumor biology and present a useful target for therapeutic interference of CML under certain circumstances.

## Results

### Expression of SOCS3 was dysregulated in CML cells

**Fig. 1:**
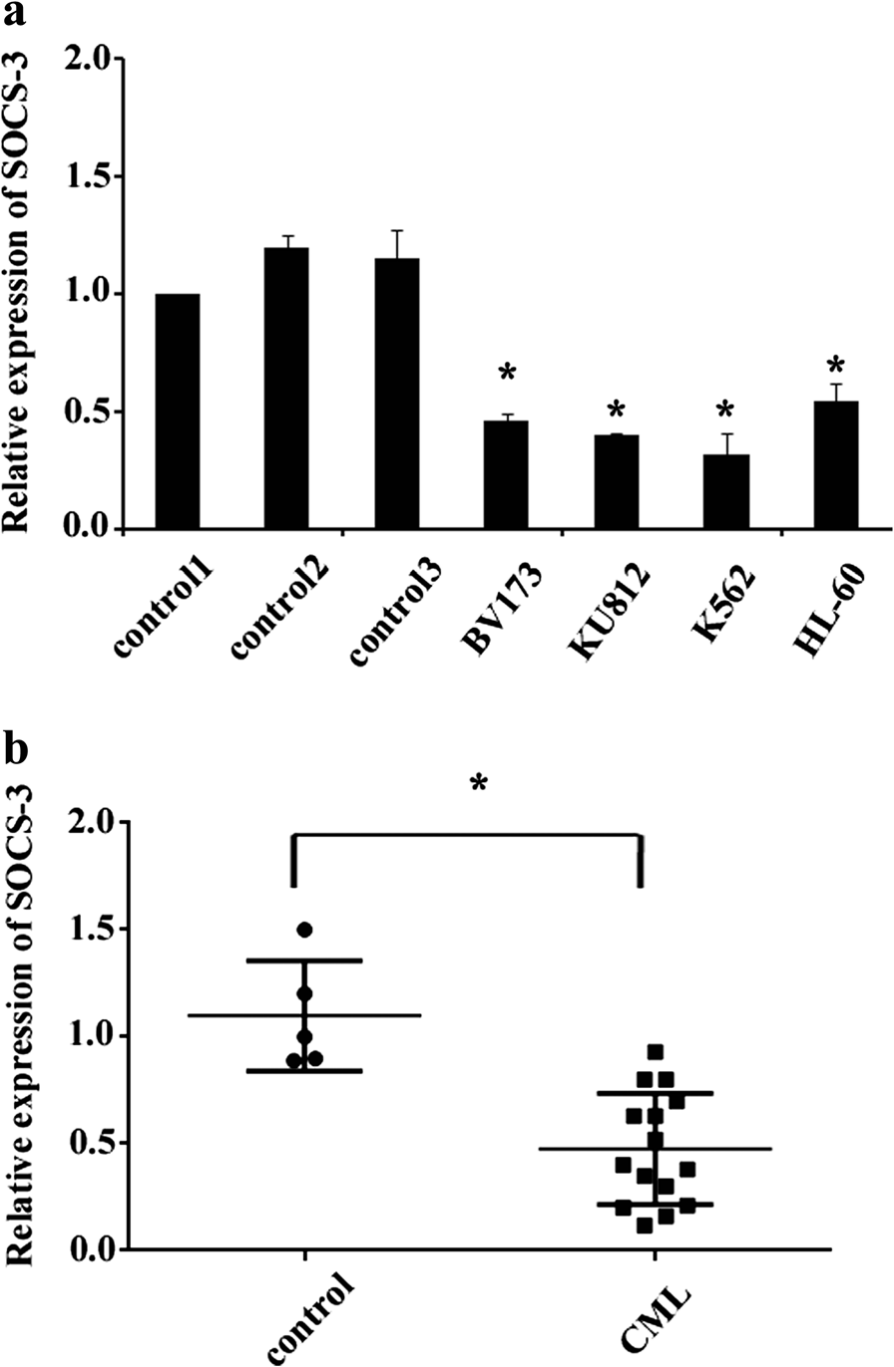
Expression of SOCS3 was dysregulated in CML cells. **a** Expression of endogenous SOCS3 was examined by q-PCR in leukemia cell lines and BMNCs from three healthy donors. β-Actin was served as an internal control. SOCS3 expression in cells from donor 1 was set as a standard. Each point represented the mean of three independent experiments (*n* = 3).*P < 0.05; compared with standard. **b** SOCS3 expression in BMNCs from five healthy donors and 15 CML patients was examined by q-PCR. β-Actin was served as an internal control. SOCS3 expression in healthy donor 1 was set as a standard. Each point represented the mean of three independent experiments (*n* = 3). *P < 0.05; compared with the healthy donor group. All data were expressed as the mean ± SD

We measured the expression of SOCS3 by qPCR in a panel of human leukemia cell lines and primary bone marrow mononuclear cells (BMNCs) from healthy donors (*n* = 3). We found that SOCS3 expression was down-regulated in human leukemia cells, with the lowest expression levels in K562 cells, indicating that SOCS3 could be down-regulated in CML (Fig. 1a). We further analyzed the expression of SOCS3 in BMNCs from 15 untreated CML patients. We found that compared with healthy donors (*n* = 5), SOCS3 expression was significantly down-regulated (which is below the minimal level of five healthy volunteers) in most of BMNCs from CML patients (12 of 15) (Fig. 1b). The protein expression of SOCS3 was also reduced greatly in CML cell lines and many patients (Additional file 1). All these findings suggested that expression of SOCS3 was dysregulated in CML cells.

### SOCS3 regulated the growth of CML cells

**Fig. 2:**
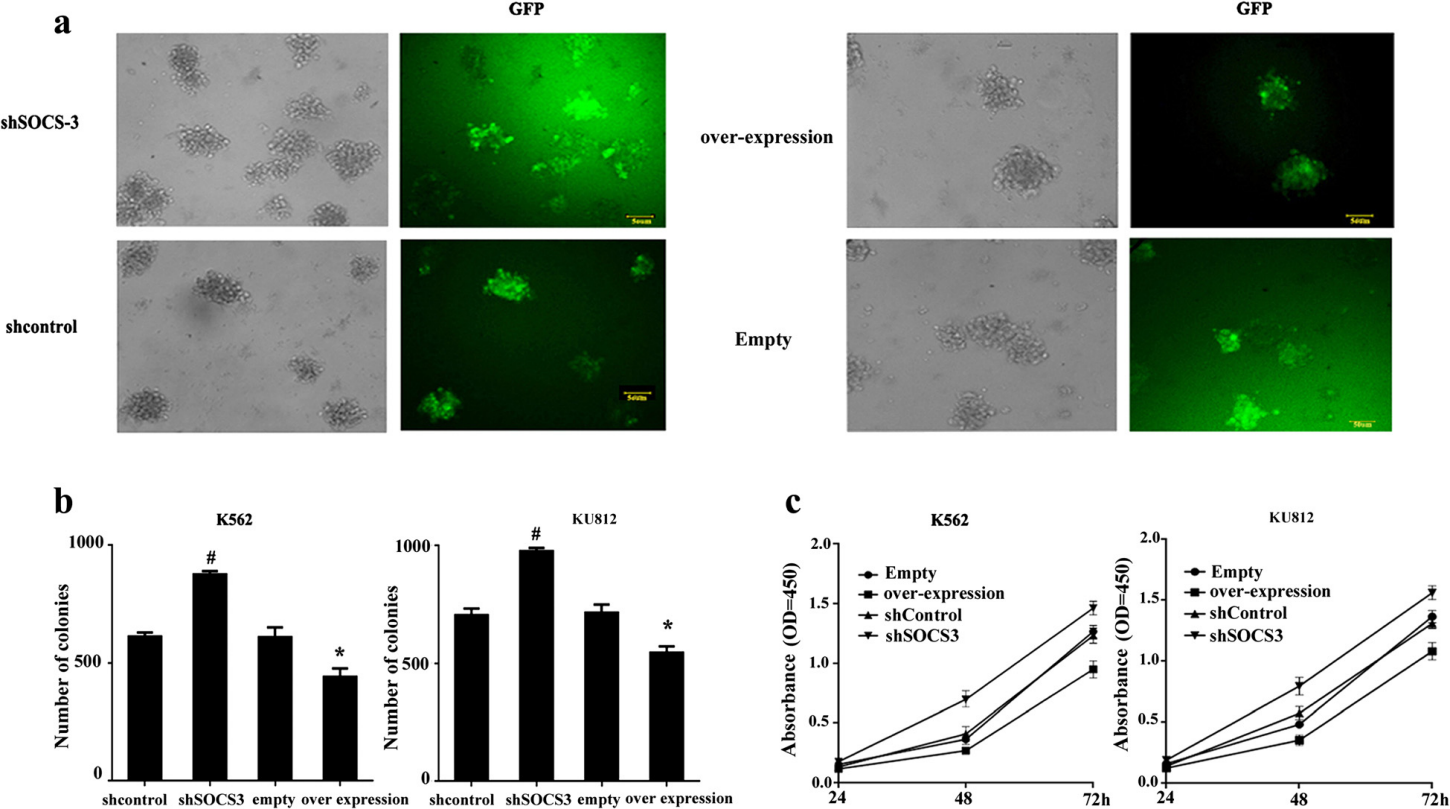
SOCS3 regulated the proliferation and clonogenic growth of CML cells. **a** Representative pictures showing the effects of SOCS3 over-expression or knock-down on the clonogenic growth of K562 cells. Bar = 50 μm. **b** Statistical analysis for the number of colonies from CML cells after over-expression of SOCS3 or shRNA vector were transduced into K562 and KU812 cells. Data represented the mean ± SD from three experiments. **c** The effect of SOCS3 on cell proliferation was examined by the CCK-8 assay. Data represented the mean ± SD from three experiments (*n* = 3). *P < 0.05; compared with the empty; ^#^ *P* < 0.05; compared with shControl

To investigate the role of SOCS3 in CML cells, the expression and inference vectors of SOCS3 were stably transduced into K562 and KU812 cells using the lentiviral system. We found that SOCS3 over-expression induced a marked reduction in the number of colonies and SOCS3 knock-down led to a significant increase in the number of colonies from K562 cells by the clonogenic formation assay (Fig. 2a, b). Similar results were observed in KU812 cells (Fig. 2b). The CCK-8 assay confirmed that SOCS3 over-expression markedly inhibited the growth of K562 and KU812 cells, while SOCS3 down-regulation promoted the proliferation of these cells when compared with empty or non-targeting shRNA (shControl) groups (Fig. 2c).

### SOCS3 contributed to imatinib-induced apoptosis of CML cells

**Fig. 3:**
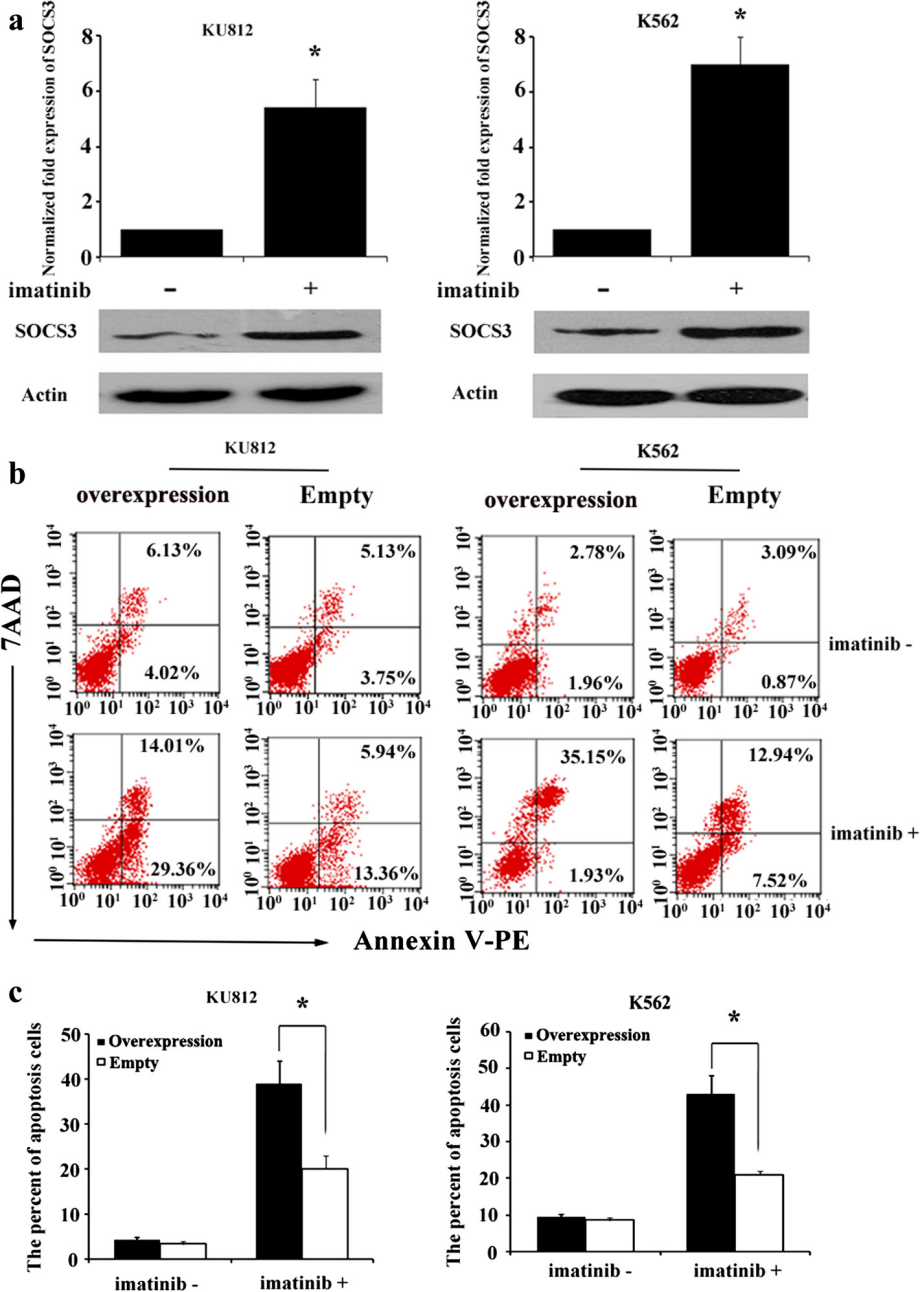
SOCS3 was up-regulated by imatinib. **a** The expression of SOCS3 in K562 and KU812 cells was examined by qPCR and western blotting after they were treated with imatinib for 48 h. β-Actin was served as an internal control. Each value was the mean ± SD of three experiments (*n* = 3), *P < 0.05; compared with untreated cells. **b** Apoptosis of K562 and KU812 cells, which were transduced with SOCS3 over-expression or empty vectors, were examined by Annexin V staining following treatment with imatinib (48 h). **c** Statistical analysis of apoptosis in K562 and KU812 cells after imatinib treatment. Data represented the mean ± SD from three experiments (*n* = 3). *P < 0.05; compared with cells transduced with empty vector

We examined SOCS3 expression levels in K562 and KU812 cells prior to and after imatinib treatment. qPCR assays demonstrated a marked increase in mRNA levels of SOCS3 after imatinib treatment in two CML cell lines. Western blot assays confirmed a marked increase in the protein levels of SOCS3 after imatinib treatment (Fig. 3a). We also found that SOCS3 itself could not induce an obvious increase in apoptotic cells when K562 or KU812 cells were transduced with SOCS3 over-expression vector. However, SOCS3 over-expression could induce a marked increase in the percentage of apoptotic cells in the presence of imatinib (Fig. 3b, c).

### SOCS3 inhibited the expression of leukemia-specific genes and promoted a series of miRNAs

**Fig. 4:**
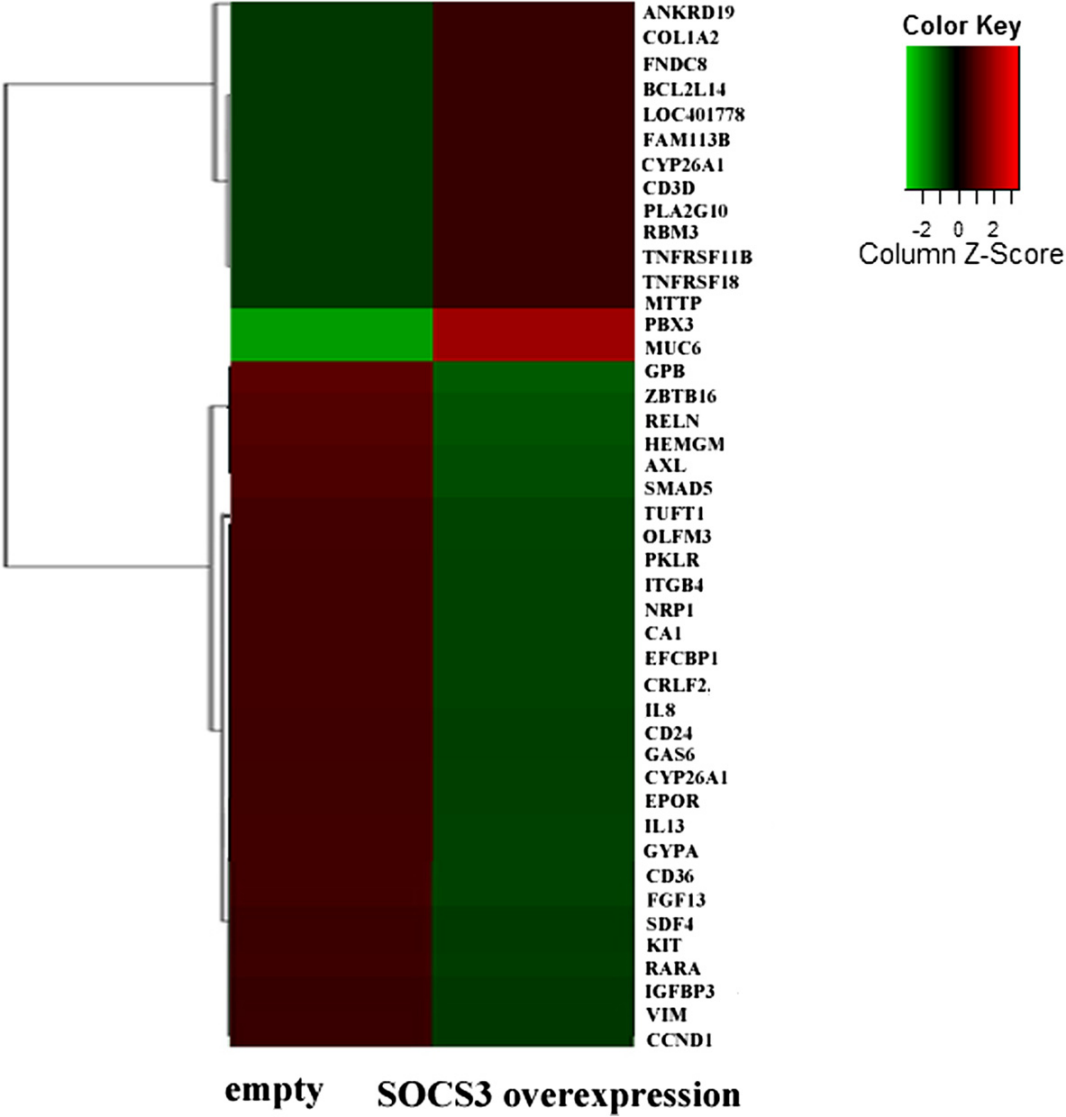
The effect of SOCS3 expression on the gene expression profile of K562 cells. Heat-map showing gene expression profiles in K562 cells 48 h after transduction with SOCS3 over-expression or empty vectors. Each kind of sample was repeated three times by microarray (*n* = 3)

To characterize the transcriptional changes caused by SOCS3, the gene expression profile of K562 was determined 48 h after SOCS3 over-expression or empty vector transduction. Total RNA from these cells was hybridized to an Illumina Human HT-12 v4 expression bead array. After normalization, the gene expression profile of two groups was compared using Benjamini Hochberg FDR values. Ultimately, we identified 296 genes with significant differential expression in K562 cells that over-expressed SOCS3. Among them, 198 genes were up-regulated and 98 genes were down-regulated. The pathways and genes that significantly changed were classified as follows: cancer pathways (*EPAS1*, *CCND1*, *FGF13*), myeloid leukemia pathways (*ZBTB16*, *KIT*, *IL13)*, hematopoietic cell lineage pathways (*EPOR*, *GYPA*, *CD36*) and so on (Fig. 4). In addition, we also found that some miRNAs were significantly affected by SOCS3 over-expression and that the expression of miR-124-3p was the highest in these miRNAs (data not shown).

Next, we investigated SOCS3-induced gene expression alteration in K562 cells using GO analysis (Additional file 2). The results confirmed that the cancer and hematopoietic cell development signaling pathways were mainly associated with responses to over-expression of SOCS3 in K562 cells.

### SOCS3 promoted miR-124-3p expression in CML cells

**Fig. 5:**
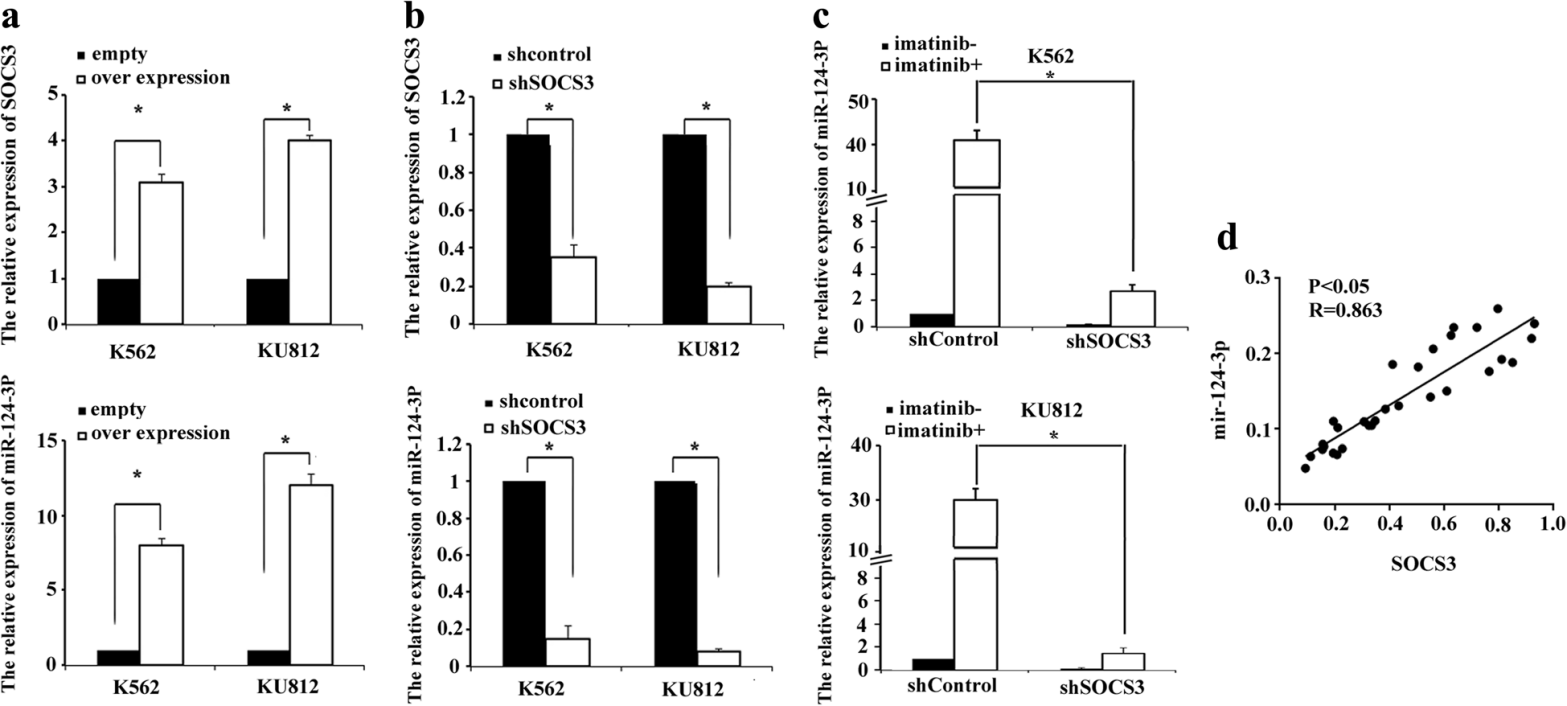
SOCS3 regulated the expression of miR-124-3p in CML cells. The relative mRNA levels of SOCS3 or miR-124-3p in K562 and KU812 cells were analyzed by q-PCR after SOCS3 over-expression (**a**) or knock-down (**b**). Each value was the mean ± SD of three experiments (*n* = 3),*P < 0.05; compared with standard. **c** Relative mRNA levels of miR-124-3p in K562 and KU812 cells that were stably transduced with shSOCS3 or shControl vector were examined at 48 h following imatinib treatment. Results were normalized to untreated cells. RNU6-2 was served as an internal control. Each value was the mean ± SD of three experiments (*n* = 3), *P < 0.05; compared with shControl. **d** Statistically significant correlation between miR-124-3p and SOCS3 expression was observed by Pearson’s method. The *y*-axis or *x*-axis represented the relative mRNA levels of miR-124-3p or SOCS3 in BMNCs from 30 CML patients, which were normalized against internal control RNU6-2 or β-Actin. Each point represented the mean of three independent experiments (*n* = 3). Data are expressed as the mean ± SD

We further confirmed the effect of SOCS3 on the expression of miR-124-3p in K562 and KU812 cell lines by q-PCR. The results showed that SOCS3 over-expression enhanced the expression of miR-124-3p, and that SOCS3 knock-down inhibited the expression of miR-124-3p in both cell lines (Fig. 5 a, b). In addition, imatinib treatment resulted in a significant increase of miR-124-3p in CML cell lines, while up-regulation of miR-124-3p induced by imatinib was inhibited by SOCS3 knock-down in K562 and KU812 cells (Fig. 5c).

Next we explored the correlation between SOCS3 and miR-124-3p in BMNCs from CML patients (*n* = 30). The levels of SOCS3 and miR-124-3p were measured and normalized. As shown in Fig. 5d, when the relative expression levels of miR-124-3p were plotted against that of SOCS3 in each patient, a significant positive correlation was found (miR-124-3p vs. SOCS3: *R* = 0.86, *P* < 0.05).

**Fig. 6:**
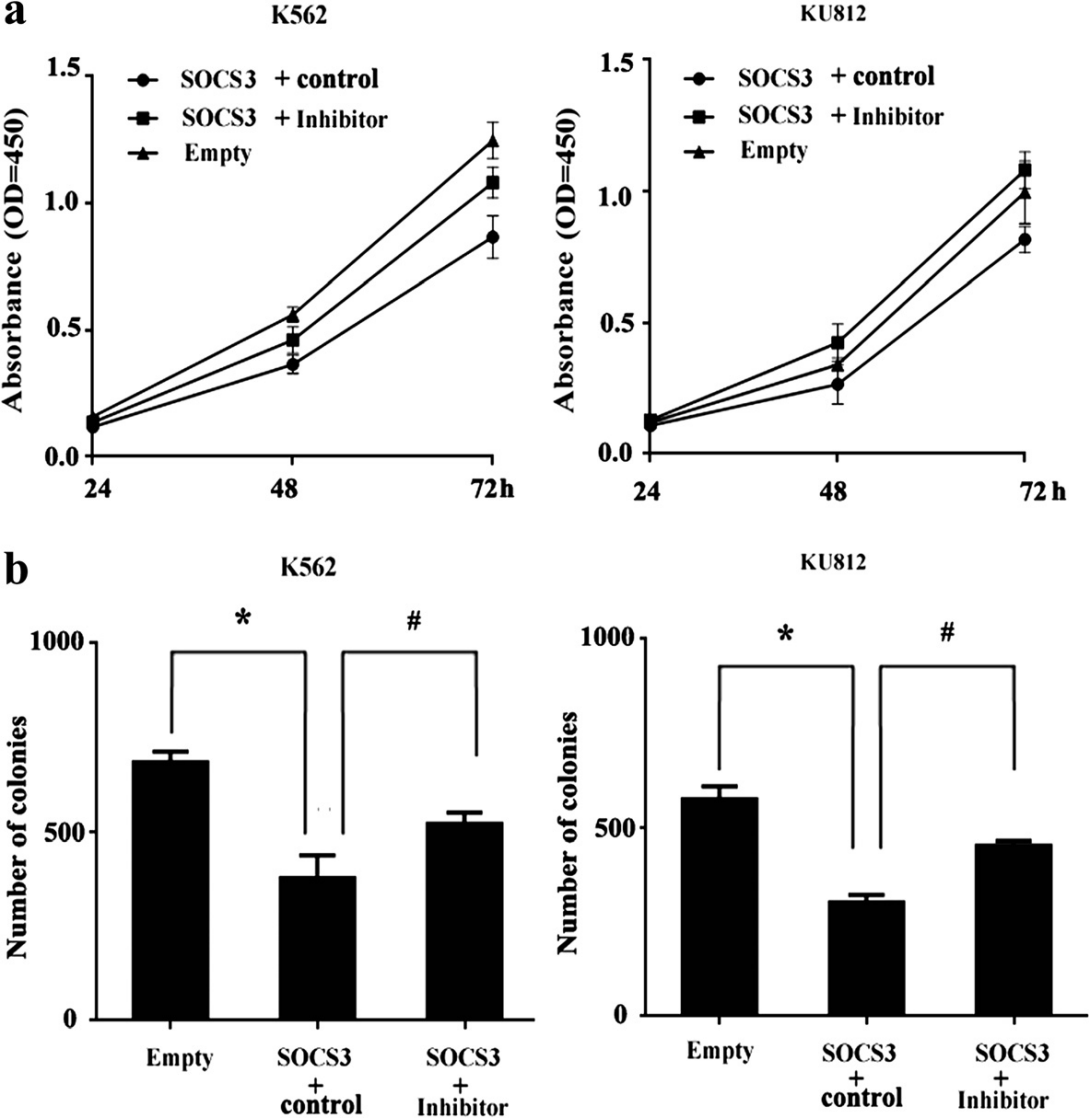
miR-124-3p inhibitor partially neutralized the inhibitory effects of SOCS3. The miR-124-3p inhibitor and control were transduced into K562 and KU812 cells that were stably transduced with SOCS3 over-expression vector. The effects of miR-124-3p on cell growth (**a**) and colony formation (**b**) were determined. Data represented three independent experiments and were shown as the mean ± SD (*n* = 3), *P < 0.05; compared with empty vector or inhibitor

We next investigated whether SOCS3 regulated CML cell function by up-regulating miR-124-3p. MiR-124-3p inhibitor and negative control were transduced into K562 and KU812 cells that were stably transduced by SOCS3 over-expression vector. Growth capacity was compared with cells transduced with empty expression vector. As expected, the cell proliferation assay and clonogenic assay showed that the miR-124-3p inhibitor partially neutralized the inhibiting effects of SOCS3 (Fig. 6).

### B4GALT1 is a target of miR-124-3p in CML cells

**Fig. 7:**
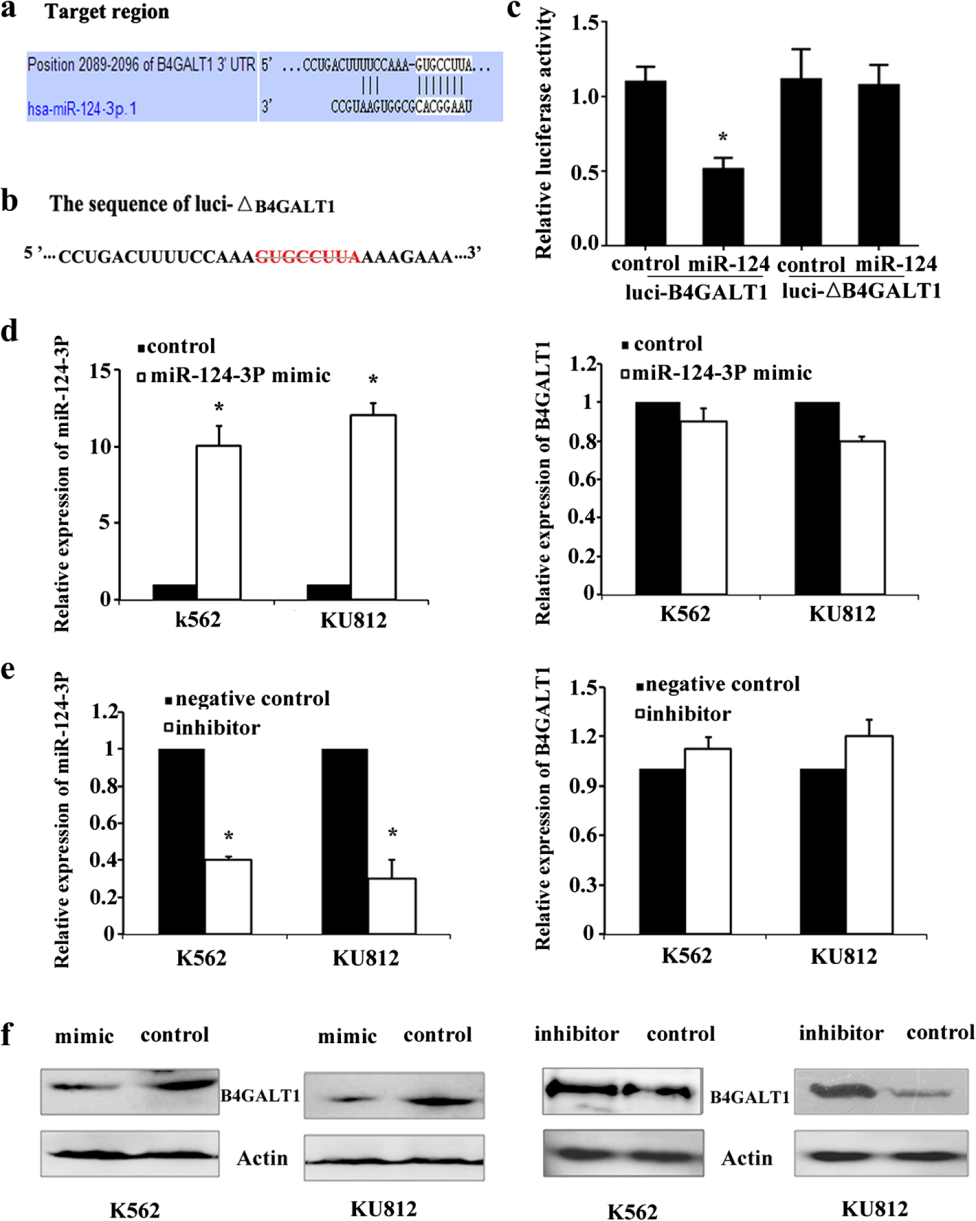
B4GALT1 was a target of miR-124-3p. **a** Bioinformatics analysis of the predicted interactions of miR-124-3p and its binding sites within the 3’UTR of B4GALT1. **b** Mutated sequences used in the luciferase assay. **c** Luciferase analysis in HEK293 cells. The expression levels of miR-124-3p or B4GALT1 in K562 and KU812 cells were analyzed by qPCR after they were transduced with miR-124-3p expression mimic (**d**) or inhibitor (**e**). U6 or β-Actin was served as an internal control. Each value was the mean ± SD of three experiments (*n* = 3),*P < 0.05; compared with control or negative control. **f** Western blot analysis of B4GALT1 expression in K562 and KU812 cells after they were transduced with miR-124-3p expression mimic or miR-124-3p inhibitor for 48 h. β-Actin was served as an internal control (*n* = 3)

Next, we searched for potential genes regulated by miR-124-3p in leukemogenesis. Using TargetScan and miRanda online search programs, we identified B4GALT1 as a potential target of miR-124-3p. A matched sequence was found at the nts 2089–2096 region of B4GALT1 mRNA 3’UTR (Fig. 7a).

To confirm that miR-124-3p targets the 3‘UTR region of B4GALT1 in CML cells, HEK293 cells were co-transduced with miR-124-3p expression or control vector along with either the full-length 3‘UTR of B4GALT1(Luci-B4GALT1) or mutated Luci-B4GALT1 reporter vectors bearing deletions of the 3‘UTR target regions (△Luci-B4GALT1) (Fig. 7b). We found that luciferase activity of HEK293 cells was significantly decreased after co-transduction of miR-124-3p expression vector and a 3‘UTR vector containing the B4GALT1/miR-124-3p target sequence (Fig. 7c).

Moreover, we over-expressed or inhibited the expression of miR-124-3p in K562 and KU812 cells and determined the endogenous expression of B4GALT1 at both the protein and mRNA level. We found that the mRNA level of B4GALT1 was not significantly affected by miR-124-3p in comparison with the control in both K562 and KU812 cells (Fig. 7d, e). However, B4GALT1 protein was markedly reduced after transduction with miR-124-3p expression vector, and vice versa (Fig. 7f). These data indicated that B4GALT1 was the target gene of miR-124-3p in CML cells, and miR-124-3p suppressed B4GALT1 gene expression at the post-transcriptional level.

### B4GALT1 was regulated by the SOCS3/miR-124-3p axis

**Fig. 8:**
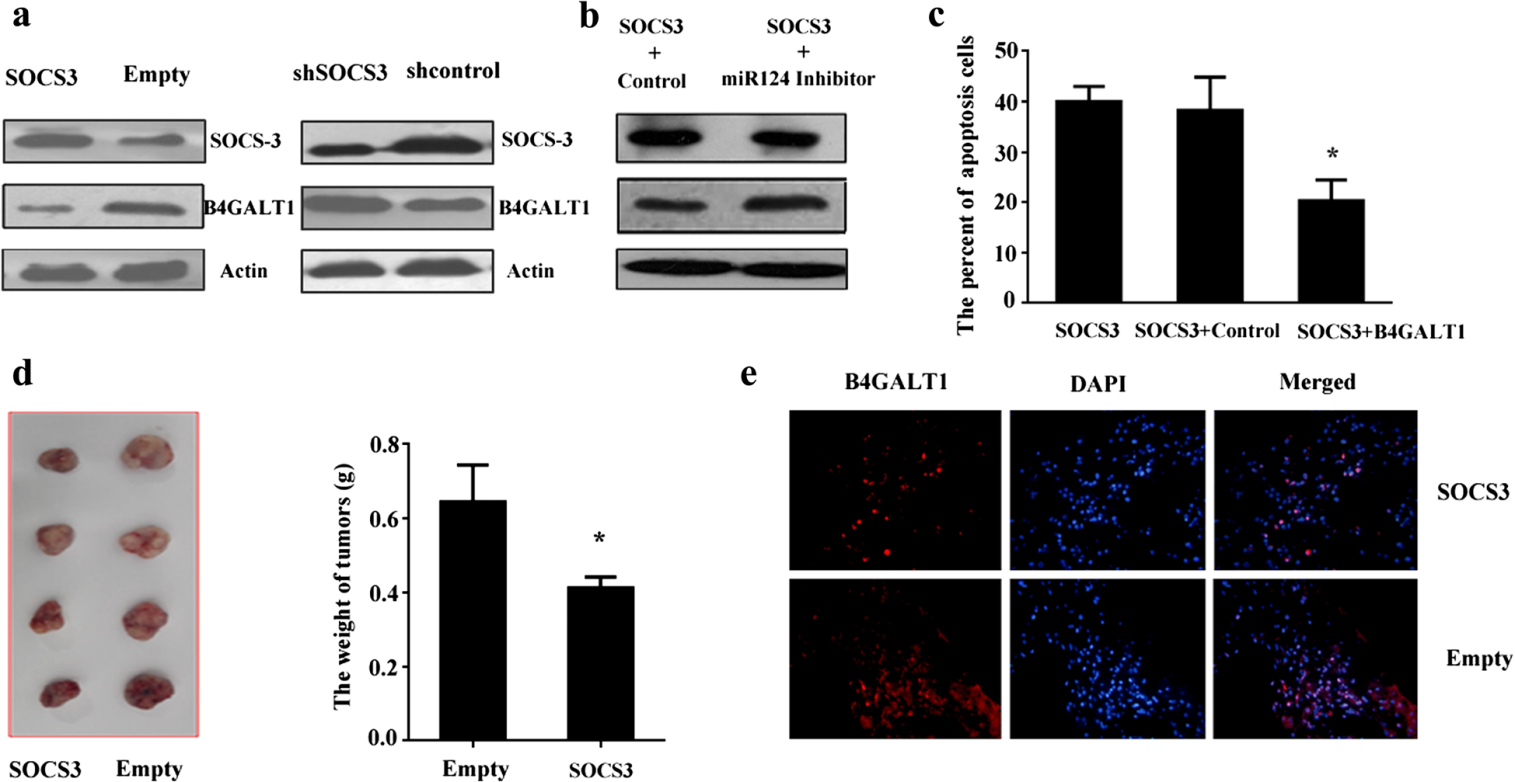
B4GALT1 was regulated by SOCS3/miR-124-3p axis. **a** Western blot analysis of B4GALT1 expression in K562 cells after SOCS3 up- or down-regulation. β-Actin was served as an internal control (*n* = 3). **b** Western blot analysis of B4GALT1 expression in K562 cells stably expressing SOCS3 after transduction with the miR-124-3p inhibitor or negative control. β-Actin was served as an internal control (*n* = 3). **c** Over-expression of B4GALT1 rescued SOCS3-induced chemo-sensitivity promotion. B4GALT1 expression and control vector were transduced into K562 cells that over-expressed SOCS3. Apoptosis ratios of K562 cells in different groups were examined by Annexin V assay after imatinib treatment. Data represent the mean ± SD from three experiments (*n* = 3). **d** SOCS3 over-expression inhibited tumor growth of K562 in vivo. *P < 0.05; compared with empty group. Each point represented the mean of three independent experiments (*n* = 3). Data are expressed as the mean ± SD. **e** SOCS3 over-expression inhibited the expression of B4GALT1 in vivo. B4GALT1 expression in tumors from K562 cells was analyzed by immunohistochemistry

The expression of B4GALT1 protein was examined in K562 cells after the expression or inference vectors of SOCS3 were stably transduced into them. The results showed that B4GALT1 expression was inhibited by SOCS3 over-expression and promoted by SOCS3 knock down in vitro (Fig. 8a). We further found that SOCS3-induced down-regulation of B4GALT1 was attenuated by the presence of the miR-124-3p inhibitor (Fig. 8b).

To investigate the impact of B4GALT1 on SOCS3-induced chemo-sensitivity promotion in leukemia cells, K562 cells which stably over-expressed SOCS3 were transduced with the B4GALT1 expression vector or its negative control and then we examined apoptosis ratios of K562 cells in different groups after imatinib treatment. We found that ectopic expression of B4GALT1 significantly abrogated increasing chemo-sensitivity induced by SOCS3 over-expression in K562 cells (Fig. 8c).

Furthermore, K562 cells (5 × 10^6^), which were transduced with SOCS3 over-expressing or empty vector, were inoculated into 25-g male nude mice subcutaneously (*n* = 8). The tumor formations were observed 4 weeks after inoculation. We found the weight of tumors in the SOCS3 over-expression group was significantly lower than that of the empty vector groups (Fig. 8d). Finally, tumor tissues were collected and sectioned. Immuno-histological examination was carried out using an antibody raised against B4GALT1. Fewer B4GALT1-positive cells were observed in the tumors of SOCS3 over-expressing group (Fig. 8e).

## Discussion

In this study, we explored the function and involved mechanisms of SOCS3 in the pathophysiology of CML. Firstly, we found that both mRNA and protein expression of SOCS3 were down-regulated in CML cell lines and most of samples from CML patients. Sakai et al. observed a large variation of SOCS3 expression in different patients [16]; here, we also found the significant difference of SOCS3 expression in different patients which implied SOCS3 may be used as an index for CMLprecision diagnosis in the future.

Previous studies demonstrated that the expression of SOCS3 was associated with the response of CML cells to IFN-alpha [16, 17], and down-regulation of SOCS3 was a possible reason for imatinib resistance of leukemia cells [11]. Consistently, in our study, the expression of SOCS3 in CML cells was up-regulated after imatinib treatment. Although SOCS3 exerted no remarkable effect on apoptosis, SOCS3 over-expression could enhance imatinib-induced apoptosis in CML cells. Takeuchi et al. suggested increased expression of SOCS3 in bone marrow cells may result from the action of several cytokines secreted in the bone marrow environment [17], so we speculated the bone marrow environment alteration caused by imatinib treatment may induce the up-regulation of SOCS3 in CML cells here. However, the in-depth mechanism needed to be clarified.

miRNAs play important roles in tumorigenesis [18–23]. We then explored whether SOCS3 regulated the growth and chemo-sensitivity of CML cells by modulating miRNA. The gene expression bead array results indicated that miR-124-3p, a tumor suppressor [24–26], was significantly affected by SOCS3. Fowler et al demonstrated that over-expression of miR-124 in GBM cells was associated with diminished tumor cell migration and invasion [27]. Moreover, Shi et al. found that miR-124-3p could inhibit the proliferation of prostate cancer cells [28]. In our study, we found that miR-124-3p expression in CML cell lines was regulated by SOCS3. A significant positive correlation between miR-124-3p and SOCS3 was observed. And, the inhibitory effect of SOCS3 on CML cell proliferation was attenuated in the absence of miR-124-3p. All these data indicated that miR-124-3p play an important role in SOCS3-mediated growth inhibition.

Recent study demonstrated that B4GALT1 gene family play an important role in the resistance of human leukemia cells to therapeutic drugs. In multidrug resistance leukemia patients, highly expressed B4GALT1 regulated the hedgehog pathway, and were associated with the expression of p-glycoprotein and multidrug resistance-associated protein, resulting in the specific drug-resistant phenotypes of leukemia cell lines [29, 30]. Here, we first demonstrated that B4GALT1 was a target gene of miR-124-3p as predicted by bioinformatics, verified the conserved region in the B4GALT1 3‘UTR was binding to miR-124-3p. In addition, we found that B4GALT1 protein expression was significantly down-regulated by miR-124-3p over-expression in CML cells. So, we speculated that SOCS3 enhanced the chemo-sensitivity of CML cells by down-regulating B4GALT1, and that miR-124-3 was the link between them. Thus, we analyzed the effect of SOCS3 on the expression of B4GALT1 and proved that SOCS3 modulated the expression of B4GALT1 by miR-124-3p and, in turn, B4GALT1 could rescue SOCS3-induced chemo-sensitivity alterations in K562 cells. Finally, tumorigenicity assays in nude mice confirmed that over-expression of SOCS3 inhibited the proliferation of K562 cells and down-regulated the expression of B4GALT1 in vivo*.* However, the in vivo function of SOCS-3 after imatinib treatment needs to be investigated in the further study.

Our results showed SOCS-3 regulated miR-124-3p/B4GALT1 pathway played an important role in the pathogenesis of CML. However, imatinib treatment still induced miR-124-3p increase in the absence of SOCS3 and SOCS3 could inhibit colony formation, regardless of the presence of miR-124-3p inhibitor, which implied other signal pathways may be involved. For example, previous studies demonstrated JAK/STAT pathway and cytokine signal pathways were involved in SOCS3-mediated effects in CML cells [16, 17].

Resistance to targeted drugs remains a challenge for CML therapy. Accurate biomarkers are of great importance to antitumor therapeutics [31]. In this study, there was an obvious correlation between SOCS3 expression and the sensitivity of CML cell lines to imatinib. Thus, SOCS3 may be used as a novel biomarker predicting the response to targeted drugs and it was of great value to further elucidate the role and mechanism underlying SOCS3 expression in CML cells. However, we did not explore the role of SOCS3 in primary cells from CML patients who are resistant to imatinib, and we acknowledge this is a limitation of this study.

## Conclusions

In summary, our work revealed that an interesting signal pathway initiated by SOCS3 was involved in CML development. Down-regulated SOCS3 in CML cells was associated with low level of miR-124-3p, then could not exert enough repressive effect on B4GALT1, resulting in the proliferation of CML cells and targeted drugs resistance. In conclusion, SOCS3/miR-124-3p/B4GALT1 signaling pathway plays an important role in the pathophysiology of CML. SOCS3 may be used as an index for CML diagnosis, and a novel biomarker predicting the response of CML to targeted drugs, in clinical settings.

## Methods

### Patient samples

BMNCs were obtained from patients with confirmed diagnose of CML and from healthy donors with informed consent. BMNCs were enriched by Ficoll gradient centrifugation. The study was performed with the approval of the Ethics Committee of Chinese PLA General Hospital, Beijing, China.

### Cell culture

K562, KU812, HL-60, and BV173 cells were cultured in RPMI-1640. HEK293 cells were cultured in DMEM. These mediums contained 10 % (v/v) fetal bovine serum (Gibco, Life technologies, USA) and 100 mg/mL penicillin/streptomycin. For imatinib treatment, cells were treated with 1 μmol/L imatinib for 48 h.

### Lentiviral plasmid construct and transduction

Lentiviral expression and interference vectors targeting human SOCS3 were constructed as described previously [12]. Empty expression vector (empty) or non-targeting siRNA (shControl) were used as controls of expression and interference vectors, respectively. Lentiviral particles were produced and cells were transduced. After transduction (48 h), positive cells were sorted by fluorescence-activated cell sorting (FACS), according to the expression of green fluorescent protein (GFP).

### Quantitative RT-PCR

Total RNA isolation, reverse transcription, and the quantification of target gene expression were performed as previously described [12]; β-Actin was used as an internal control. miR-124-3p expression levels were quantified using U6 as the internal control (GenePharm). The fold-change in expression was calculated using the following primer pairs for the amplification of target mRNAs: SOCS3 forward primer 5‘ATCCTGGTGACAT GCTCCTC’3 and reverse primer 5‘CAAATGTTGCTTCCCCCTTA’3; β-Actin forward primer 5‘ GATCCACATCTGCTGGAA GG’3 and reverse primer 5‘AAGTGTGACGTT GACATCCG’3; B4GALT1 forward primer 5‘AACCATGTGACTGAGTGC CC’3 and reverse primer 5‘TCAGTGTGTTGTGCCAAAGC’3; Micro124-3p forward primer 5‘TAAGGCACGCGGTGAATGCC’3 and reverse primer 5‘GATTGAATCGA GCACCAG TTAC’3; U6 forward primer 5‘CGCTTCGGCAG CAC ATATACTA’3 . Unified reverse primer 5‘GATTGAATCGA GCACCAG TTAC’3.

### Western blot analysis

Cells were lysed directly in lysis buffer to collect whole cell extracts. Protein samples were separated on polyacrylamide gels, transferred onto nitrocellulose membrane by iblot (Invitrogen), detected using horseradish peroxidase-conjugated secondary antibodies, and exposed to BioMax film (Kodak) following chemiluminescence (Santa Cruz, CA, USA). The following primary antibodies were used: SOCS3, Actin, and B4GALT1 (Santa Cruz, CA, USA).

### Cell proliferation assay

Cell proliferation was determined using CCK-8 (Dojindo, Japan) method. K562 cells (3000 cells/well) were plated in 96-well plates. At different time points, CCK-8 reagents were added to each well and further incubated at 37 °C for 2 h. The number of viable cells was assessed by measurement of absorbance at 450 nm using a Multiskan (Thermo Scientific, Asheville, NC, USA).

### Clonogenic progenitor cell assay

Cells were seeded in a 6-well plate with methylcellulose medium (MethoCult H4435, STEMCELL Technologies, Canada) according to the manufacturer’s instructions. After 1 week of cultivation, colonies were counted.

### Apoptosis assay

The apoptosis assays were performed using the Annexin V-PE kit (BioLegend, San Diego, CA, USA) according to the manufacturer’s protocol. The stained cells were immediately analyzed on a FACScalibur flow cytometer (Becton Dickinson). The data were expressed as the percentage of apoptosis cells.

### Whole-genome expression analysis

Total RNA was extracted from K562 cells transduced with over-expression SOCS3 or empty vector (5 × 10^6^ cells) using Trizol reagent (Invitrogen Life Technologies, Paisley, UK). Genome expression analysis was performed by Illumina Human HT-12 v4 BeadChip (Illumina, San Diego, CA, USA) at the Beijing Qian zhao xing ye Biological Technology Co., Ltd. (Beijing, China).

### miR-124-3p mimic and inhibitor

The hsa-miR-124-3p mimic or control sequence, and hsa-miR-124-3p inhibitor and hsa-miR-124-3p inhibitor negative control were all purchased from GenePharma (Shanghai, China).

### Luciferase assays

The human pre-miR-124 sequence was amplified and cloned into pcDNA3.1 constructs (Invitrogen) to generate the pcDNA3.1-miR-124 expression vector. The full-length 3‘UTR of B4GALT1 was amplified using cDNA from K562 cells and double-digested with XbaI/EcoRI and cloned downstream of the firefly luciferase coding region sites of a modified pGL3-control plasmid named Luci-B4GALT1. We also constructed mutated Luci-B4GALT1 reporter vectors bearing deletions of the UTR target regions and named them Luci-△B4GALT1. The vectors were co-transduced with control or pcDNA3.1-miR-124 expression vectors into K562 cells. Lysates were prepared 48 h after transduction. Luciferase activity was measured using a dual-luciferase reporter assay system (Promega). All experiments were performed in triplicate at least three independent times.

### B4GALT1 expression plasmid construction and transduction

The full-length of the human B4GALT1 sequence was amplified and cloned into the pcDNA3.1 vector to generate the B4GALT1 expression vector. K562 cells were transduced with the B4GALT1 expression vector in 24-well plates using Lipofectamine 2000 (Invitrogen) according to the manufacturer’s protocol.

### Tumorigenicity assays in nude mice

A total of 5 × 10^6^ K562 cells that were stably transduced with SOCS3 over-expression or empty vectors were injected into male nude mice subcutaneously (*n* = 8). Mice were sacrificed at 28 days post-inoculation, and the tumors were excised and their weight was measured and photographed. Tumors from different groups were removed and fixed with formaldehyde, embedded in paraffin wax, and sectioned. The sections were cleared through xylene, graded ethanol, water and incubated with anti-B4GALT1 antibodies (Santa Cruz) at 4 °C overnight, stained with DAPI, washed with PBS three times, and observed by fluorescence microscopy.

### Statistical analysis

All data were expressed as the mean ± standard deviation (SD). The differences between two groups were assessed by Student’s *t* test. Pearson’s correlation coefficient was calculated to analyze the correlation. *P* < 0.05 was considered to be statistically significant.

## Abbreviations

3’-UTR, 3’-untranslated region; BMNCs, bone marrow mononuclear cells; CML, chronic myeloid leukemia; CMPD, chronic myeloproliferative disorders; FACS, fluorescence - activated cell sorting; GFP, green fluorescent protein; miR-124-3p, microRNA-124-3p; miRNAs, microRNAs; qPCR, quantitative real-time PCR; siRNA, small interfering RNA; SOCS3, suppressor of cytokine signaling 3; WB, Western blotting

## Additional files

Additional file 1: Figure S1. Expression of SOCS3 protein in CML cells. SOCS3 expression in CML cell lines and BMNCs from CML patients was analyzed by Western blotting. β-Actin was served as an internal control (*n* = 3). (TIF 69 kb) Additional file 2: Figure S2. GO analysis of SOCS3-induced gene expression alteration in K562 cells. Branches of the GO hierarchical tree with significantly enriched GO terms were indicated in red boxes. Insignificant GO terms within the hierarchical tree are shown as white boxes. (TIF 203 kb)
