# Supplementary figures and images for: MiR-124-3p/B4GALT1 axis plays an important role in SOCS3-regulated growth and chemo-sensitivity of CML
Source: J Hematol Oncol. 2016 Aug 12;9:69. doi: 10.1186/s13045-016-0300-3 (PMC4982324; doi:10.1186/s13045-016-0300-3)

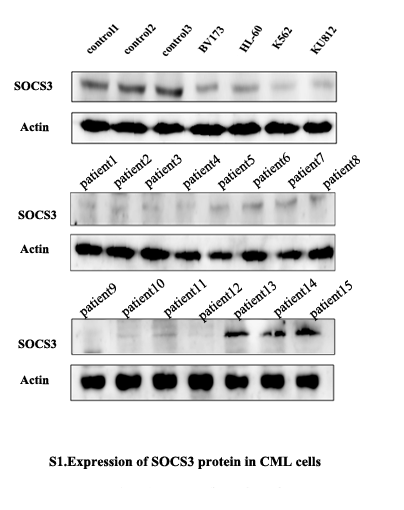

Supplement: Additional file 1: Figure S1. — Expression of SOCS3 protein in CML cells. SOCS3 expression in CML cell lines and BMNCs from CML patients was analyzed by Western blotting. β-Actin was served as an internal control (n = 3). (TIF 69 kb) [file 13045_2016_300_MOESM1_ESM.tif]

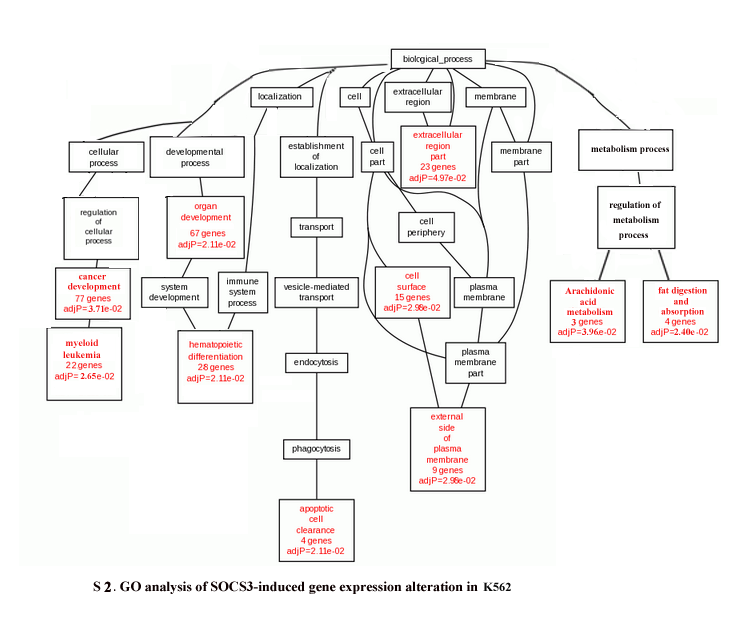

Supplement: Additional file 2: Figure S2. — GO analysis of SOCS3-induced gene expression alteration in K562 cells. Branches of the GO hierarchical tree with significantly enriched GO terms were indicated in red boxes. Insignificant GO terms within the hierarchical tree are shown as white boxes. (TIF 203 kb) [file 13045_2016_300_MOESM2_ESM.tif]
